# Durable Response and Good Tolerance to the Triple Combination of Toripalimab, Gemcitabine, and Nab-Paclitaxel in a Patient With Metastatic Pancreatic Ductal Adenocarcinoma

**DOI:** 10.3389/fimmu.2020.01127

**Published:** 2020-06-19

**Authors:** Lin Shui, Ke Cheng, Xiaofen Li, Pixian Shui, Shuangshuang Li, Yang Peng, Jian Li, Fengzhu Guo, Cheng Yi, Dan Cao

**Affiliations:** ^1^Department of Abdominal Oncology, State Key Laboratory of Biotherapy, Cancer Center, West China Hospital, Sichuan University, Chengdu, China; ^2^School of Pharmacy, Southwest Medical University, Luzhou, China; ^3^Department of Breast Surgery, The First Affiliated Hospital of Chongqing Medical University, Chongqing, China; ^4^Department of Pharmacy, The Affiliated Traditional Chinese Medicine Hospital of Southwest Medical University, Luzhou, China; ^5^State Key Laboratory of Biotherapy, Lung Cancer Center, Cancer Center, West China Hospital of Sichuan University, Chengdu, China

**Keywords:** PD-1 inhibitor, chemotherapy, combination therapy, metastatic pancreatic ductal adenocarcinoma, durable response, good tolerance, case report

## Abstract

**Background:** The performance of immune checkpoint inhibitor (ICI) monotherapy was proved to be disappointing in pancreatic ductal adenocarcinoma (PDAC). Increasing evidence has shown the promising efficacy of ICIs combined with systemic therapy in the first-line treatment in solid tumors.

**Case presentation:** We reported a case of a metastatic PDAC patient who had a long-term partial response and good tolerance to the combined approach of toripalimab (a novel PD-1 inhibitor) and gemcitabine plus nab-paclitaxel (GA). PD-L1 positive expression was detected in his liver metastases. Besides, we described a phenomenon of pseudo-progression of this patient during the course of therapy.

**Conclusion:** As the first-line treatment of metastatic PDAC patients, GA plus toripalimab may provide a novel combined approach with favorable response and manageable toxicity. Further clinical trials are needed to confirm the results. Pseudo-progression requires special attention and to be differentiated with true progression in patients undergoing immunotherapy.

## Background

Metastatic pancreatic ductal adenocarcinoma (PDAC) is one of the most fatal diseases with increasing incidence and mortality. Between 2009 and 2016, the 5-year survival rate for PDAC fluctuated <9% (1). Insufficient selections are efficacious in this refractory disease due to its poor response. Since the MPACT trial indicated prolonged overall survival in first-line treatment of gemcitabine plus nab-paclitaxel (GA) compared to gemcitabine alone (2, 3), GA has substituted gemcitabine as the standard of care at the expense of the high possibility of side effects (4). Therefore, GA was recommended as the first choice to metastatic PDAC patients with Eastern Cooperative Oncology Group performance status (ECOG PS) 0 to 1, as well as on the condition of patients' preference and available support system (5). Despite some attempts of novel regimen, significant improvement in clinical outcomes of PDAC patients has remained absent.

Recently, immune checkpoint inhibitors (ICIs) have been approved in patients with mismatch repair-deficient (dMMR) (6) or microsatellite instability-high (MSI-H), irrespective of which types of tumor (7). Unfortunately, the success of ICIs has not been replicated in PDAC: no objective response was observed in either anti-PD-1/PD-L1 antibody or anti-CTLA-4 (cytotoxic T lymphocyte antigen-4) monotherapy in any research (8, 9). Plausible explanations contributing to poor efficacy of ICIs in PDAC mainly involve the tumor cell-intrinsic characteristics, including the low immunogenicity, such as low mutational burden and fewer neoantigens, as well as the prominent desmoplastic stroma surrounding PDAC tumors, which may impede the ability of CD8^+^ T effector cells to infiltrate into the tumor to exert their killing effect.

Herein, we report a case of a metastatic PDAC patient with high PD-L1 expression who had a partial response and good tolerance to combination of toripalimab, a novel PD-1 blockade, and GA chemotherapy. We also review relevant literature about combination therapy of ICIs and chemotherapy in PDAC.

## Case Presentation

**Figure 1:**
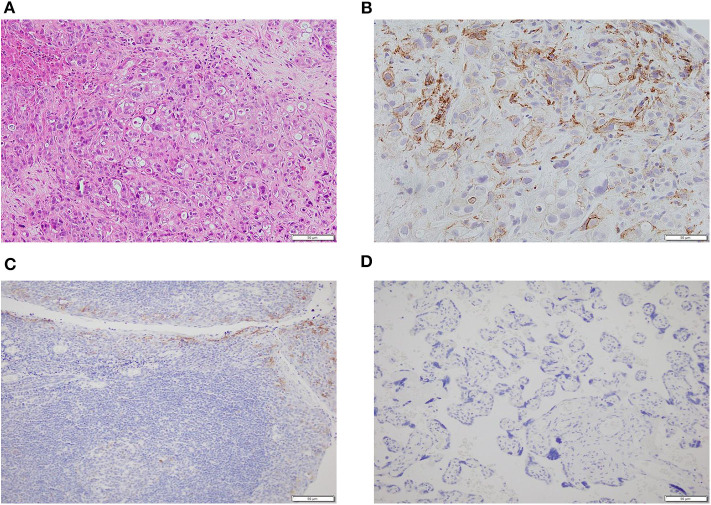
The histopathology and immunohistochemistry (IHC) of metastatic tumor tissues of this patient. **(A)** The H&E staining in the microscopic observation (100×). **(B)** Immunohistochemical staining for PD-L1 expression (400×) showed that the tumor cells were positive for PD-L1. **(C)** The positive control of the IHC of PD-L1 expression (200×). **(D)** The negative control of the IHC of PD-L1 expression (200×).

A 58-year-old man was found with some liver lumps by abdominal ultrasonography in his regular physical check-up in May 2019. Without any symptoms before, he went to the hospital for further examination. A test of tumor markers showed that serum CA125 was 1,898 U/ml and the CA199 level was out of the upper limit of detection (>1,000 U/ml). A computed tomography (CT) scan and magnetic resonance imaging (MRI) of the abdomen both indicated multiple liver lesions and a pancreatic tail mass at a size of 3.9 × 2.6 cm. He was referred to the Department of General Surgery and underwent a laparoscopic liver biopsy. Intraoperative findings showed multiple scattered nodules on the surface of the liver, whose diameters were <2 cm. Pathology showed metastatic ductal adenocarcinoma. Given these findings, his final diagnosis was pancreatic adenocarcinoma with multiple liver metastases (cT_2_N_+_M_1_, stage IV). The next-generation sequencing of his tumor showed an intermediate tumor mutation burden with 5.65 mutations/megabase and microsatellite stable (MSS) status. The immunohistochemistry (IHC) data of the tumor tissue of this patient indicated the positive expression of PD-L1 protein (30%), and the tumor proportion score (TPS) was 20% and combined positive score (CPS) was 30 (Figure 1). Additionally, deleterious alterations occurred in CDKN2A, KRAS, TP53, and VEGFA genes. There were not any applicable targeted drugs for these gene mutations.

**Figure 2:**
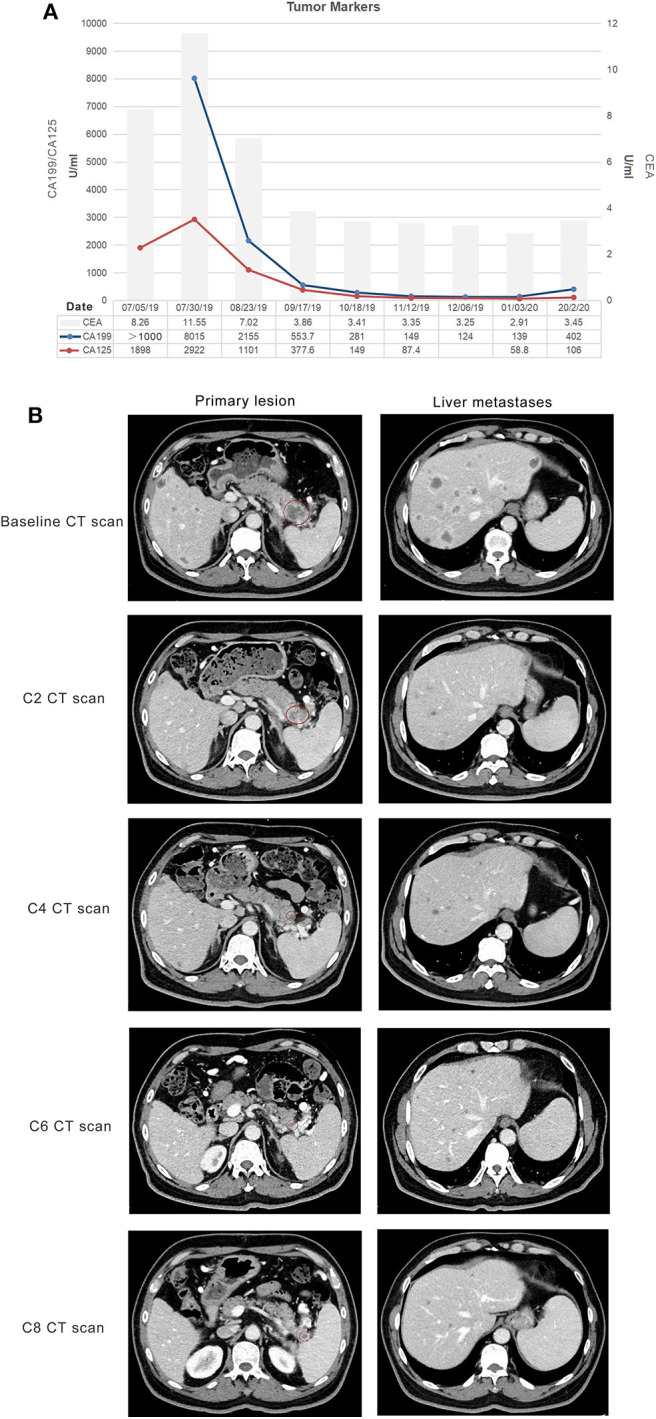
Response evaluation during the clinical course. **(A)** Trends in the level of tumor markers, including CA 199, CA 125 (left *Y*-axis), and CEA (right *Y*-axis) corresponding to the treatment timeline. *X*-axis showing the date of the disease course. The loss of the first value of CA 199 was due to the out of range of detection. **(B)** Representative images of the CT scan showed that both primary and metastatic lesions were shrunk and decreased after two cycles of gemcitabine plus nab-paclitaxel combined with a PD-1 antibody (toripalimab). Red circles indicate the primary pancreatic lesions.

With his content, he was eligible for a clinical trial about the combination of doublet chemotherapy (gemcitabine 1,000 mg/m^2^ and nab-paclitaxel 125 mg/m^2^) and toripalimab (a novel PD-1 inhibitor, 240 mg) for the first-line treatment of metastatic PDAC conducted by our department. Therefore, he received gemcitabine 1,700 mg and nab-paclitaxel (Abraxane) 200 mg at day 1 and day 8, along with toripalimab 240 mg at day 1 every 3 weeks. After 2 cycles of the combination therapy, his metastatic liver lesions almost disappeared with an evaluation of partial response (PR) by Response Evaluation Criteria in Solid Tumors (RECIST) version 1.1 (Figure 2). Surprisingly, he did not suffer any serious side effects except mild nausea and loss of appetite (grade 1, CTCAE 3.0), which was self-cured at the interstitial period of therapy. The treatment continued and repeated CT scans after four cycles showed shrinkage of the primary lesion but an increase in the number of the liver metastases. However, the CA199 level plummeted from 8,015 to 553.7 U/ml after the first four cycles of treatment (Figure 2). He was still asymptomatic but had grade 1 myelosuppression, which was successfully treated with a recombinant human interleukin-11. Through multidisciplinary therapy (MDT) and communication with the patient, we thought that it was highly possible for him to have radiological pseudo-progression and suggested he continue the therapy regimen. As we expected, the subsequent two-cycle treatment brought new clinical benefits to this patient, which in turn confirmed the previous diagnosis of pseudo-progression. The patient's continuous PR is still ongoing at the time of this report (eight cycles after the initial of the combination therapy). Primary and metastatic lesions were significantly decreased or shrank to nearly invisible status as the last evaluation showed, and the level of CA 199 has maintained within the normal for a long period but a little increase at the last test (Figure 2). All treatment-related adverse events (TRAEs) of this patient throughout the clinical course were listed in Table 1. The most serious TRAEs he had was grade 2 leukocytopenia, which was recovered under drug intervention before the next cycle treatment. Overall, he did not suffer any grade 3 or higher toxicities and maintained good tolerance. With a history of hypertension and type II diabetes, the patient also kept his blood pressure and blood glucose under good control.

**Table 1:** Hematologic and non-hematologic adverse events in the therapeutic course presented in this patient, which were graded using CTCAE 3.0.

**Hematologic adverse**	**Baseline**	**Maximum grade during**	**Non-hematologic adverse**	**Baseline**	**Maximum grade during**
**events**		**treatment**	**events**		**treatment**
**CTCAE grades**					
Leukocytopenia	0	II	Nausea	0	I
Thrombocytopenia	I	I	Pruritus	0	I
Hypohemia	0	I	Poor appetite	0	I

*CTCAE, Common Terminology Criteria for Adverse Events*.

## Discussion

Many cases of exceptional or durable responses to ICIs have been reported. To our knowledge, however, this is the first report showing the striking long-term response and safety of doublet chemotherapy combined with toripalimab in the first-line treatment of PDAC.

Toripalimab is the first recombinant humanized anti-PD-1 monoclonal antibody which was independently developed by Chinese companies. It was approved by the National Medical Products Administration (NMPA) of China in December 2018 for locally advanced or metastatic melanoma after systemic treatment failure. It has a high binding affinity, which enables it to bind its specific antigen PD-1 receptor more firmly and compete better with PD-L1 and PD-L2 binding on tumor cells. After binding, it can induce strong endocytosis of PD-1 receptor, thus reducing the expression of PD-1 on the cell membrane surface. A study revealed the different binding orientation of toripalimab compared to other PD-1 blockade, which binds PD-1 mainly on a loop that contributes multiple interactions with PD-L1 (10). The distinct biomolecular characteristics of toripalimab might result in different properties. Recently, increasing studies about various malignancies has proven the potential superiority of toripalimab, especially good tolerability, which may provide an opportunity to use concurrently with other anti-tumor drugs (11). In a phase II study, toripalimab combined with capecitabine and oxaliplatin (CapeOX) as the first-line treatment to treat patients with advanced gastric cancer, and the overall response rate (CR and PR) was 66.7% and the disease control rate (CR, PR, and SD) was 88.9%. Besides, nearly 38.9% of patients experienced grade 3 or 4 TRAEs (12). Compared to the ATTRACTION-4 trial, the encouraging efficacy of toripalimab was not inferior to Nivolumab with an ORR of 76.5%, but much more grade 3 or greater TRAEs occurred in 66.7% of patients with Nivolumab plus CapeOX (13).

This patient may not be sensitive to PD-1 blockade according to ASCO clinical practice guideline, which approved PD-1 blockade for patients with dMMR (6) or MSI-H (14). Given the predictive role of PD-L1 overexpression in PDAC was still controversial, our case suggested that PD-L1 overexpression may have the potential to select population. Moreover, emerging evidence supported combining systemic therapy on an ICIs backbone to overcome resistance due to the superior safety of ICIs (15). Theoretically, systemic chemotherapy was regarded as an immunogenic approach by stimulating anti-cancer immune effectors or inhibiting immunosuppressive factors (16). It may increase the expression or presentation of tumor-associated antigens on the surface of cancer cells, inducing signal emission to trigger immune response. As a method for priming the quiescent tumor microenvironment, chemotherapy has the potential to potentiate immunogenicity and antigenicity of tumors, thus enhancing the likelihood of recognition and killing of tumor cells by immune effector (17). For example, gemcitabine may upregulate the expression of class I human leukocyte antigen and promote the cross-presentation of tumor antigen, therefore selectively eliminating myeloid-derived suppressor cells (MDSCs) to overcome the immunosuppression. Paclitaxel was proved to stimulate antigen-presenting cells and improve the release of granzyme B by effector cells (18). Some phase I/II studies have confirmed the synergetic effects of cytotoxic chemotherapy with ICIs in other types of cancer (18–20). There are limited data about the safety and efficacy of combination of ICIs and chemotherapy in metastatic PDAC (Table 2). Results from a phase Ib/II study conducted in metastatic PDAC suggested that the efficacy of combined chemo-immunotherapy appears to be slightly improved over conventional standard chemotherapy (27). Others have highlighted the importance of combination therapy in the first-line treatment to obtain initial remission. The impressive results of this case need to be further confirmed by a large-scale randomized controlled study.

**Table 2:** Efficacy and safety of combined therapeutic approaches of immune checkpoint inhibitors and chemotherapy in pancreatic cancer.

**References**	**Phase**	**No. of patients**	**Disease**	**Treatment**	**Response**	**Adverse events**
Weiss et al. (18)	Ib/II	17	Metastatic 1st line	Gemcitabine + Nab-Paclitaxel + Pembrolizumab	25% PR; 67% SD	Any grade of TRAEs: all (100%); Grade 3 or 4 TRAEs: 12 patients (70.6%)
Renouf et al. (21)	II	11	Metastatic 1st line	Durvalumab + tremelimumab + gemcitabine + nab-paclitaxel	PR 8/11 (73%); DCR (100%); Median PFS 7.9 months	Grade 3 or greater TRAEs: fatigue (27%), anemia (36%), abnormal WBC (27%), hyponatremia (27%), hypoalbuminemia (45%), abnormal lipase (45%). colitis (9.1%)
Wainberg et al. (22)	I	50	Locally advanced/ Metastatic 1st line	Nivolumab + nab-paclitaxel + gemcitabine	2% CR; 16% PR; 46% SD	Grade 3 or 4 TRAEs: 48 patients (96%)
Borazanci et al. (23)	II, pilot	11	Metastatic 1st line	Nivolumab + nab-paclitaxel + cisplatin + gemcitabine + paricalcitol	80% PR; 100% DCR	Grade 3 or 4 TRAEs: thrombocytopenia 76%, anemia 44%, colitis 12%
Aglietta et al. (24)	Ib	34	Metastatic 1st line	Tremelimumab + gemcitabine	PR 2/19 (10.5%)	Any grade TRAEs: 12 pts (35.3%); grade 3 or 4 TRAEs: 2 pts (5.9%)
Kamath et al. (25)	Ib	21	Gemcitabine-naïve	Ipilimumab + gemcitabine	ORR 14%; PR 2/16 (12.5%)	Grade 3 or higher TRAEs: 16 pts (76%)
Wainberg et al. (26)	I	17	1st and 2nd line	Arm A: Nivolumab + nab-paclitaxel (2nd line) Arm B: Same as arm A with gemcitabine (1st line)	Arm A: PR 2/9 (22.2%) SD 4/9; Arm B: PR 3/6 (50%) SD 3/6	Grade 3/4 TRAEs: Arm A: 2/11 pts (18%); Arm B: 2/6 pts (33%)

*CR, complete response; PR, partial response; SD, stable disease; PD, progressive disease; TRAEs, treatment-related adverse events; Pts, patients*.

**Figure 3:**
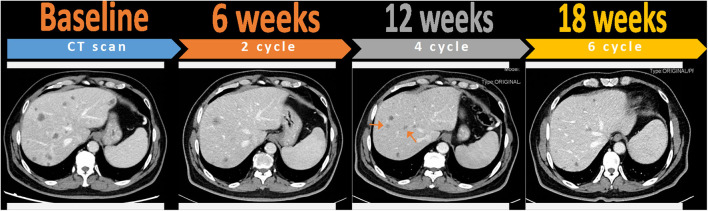
Pseudo-progression of liver metastases after four cycles of toripalimab combined with gemcitabine and nab-paclitaxel, which was confirmed by favorable outcomes at the next assessment of six cycles. Red arrows indicate the appearance of new lesions that were invisible at previous CT evaluation and then disappeared after the continuation of therapy.

Interestingly, the patient presented rare pseudo-progression on the CT scan in the treatment course. Pseudo-progression is defined as temporarily enlarging lesions or the appearance of new lesions detected by imaging tests undergoing cancer immunotherapy (28). As the term suggests, pseudo-progression is not a real progression of the disease, whereas it may be linked with a durable response to immunotherapy (29). Presentation of pseudo-progression may be explained as edema and necrosis of tumor tissues caused by the infiltration of immune cells (30), resulting in morphologically similar mass around the original lesions in the imaging. Besides, with the characteristics of a late response, immunotherapy may not induce tumor regression until CT evaluation after the next few cycles of treatment. Instead of treatment failure, this kind of transient tumor growth before the onset of immune response needs to be distinguished with the real progression. Exaggerating the occurrence of pseudo-progression is not advisable because over-treatment may damage life quality, especially for metastatic cancer patients whose main purpose is to alleviate their symptoms. In this case, the patient was found to have new liver lesions after four cycles of treatment (Figure 3). Given he was not accompanied by clinical deterioration and the continuously falling CA-199 level, we inferred that the emergence of new lesions may result from the pseudo enlargement of small lesions that were invisible on the baseline CT scan. As we expected, the newly presented lesions disappeared and original lesions shrank after the continuation of this therapy, which verified our diagnosis of pseudo-progression.

Besides impressive efficacy, he also had a good tolerance to these triple anti-tumor drugs, especially PD-1 inhibitors whose immune-related adverse events need special attention. After the first 2 cycles of therapy, he encountered grade 1 myelosuppression, which was successfully treated with a recombinant human interleukin-11, along with a self-cured gastrointestinal tract reaction. Subsequently, he experienced no more overt toxicities and was well-tolerated to a total of 8 cycles of combination therapy. Taking concurrent anti-hypertensive drugs and metformin with this highly intensive anti-tumor regimen, his liver and renal function were still within the normal range.

In summary, combined therapy of toripalimab and standard chemotherapy is potentially effective and well-tolerated as the first-line treatment in metastatic PDAC. Although the data are limited to conclude, we presented a patient who had a striking response to this combination as well as a manageable safety profile. The favorable clinical outcome may be attributed to safer toripalimab or the synergistic function of chemotherapy and PD-1 blockade. Furthermore, this case also displayed the possibility of the phenomenon of pseudo-progression under this regimen, which needed to be taken into consideration in the design and process of clinical trials.

## Ethics Statement

Written informed consent was obtained from the individual(s) for the publication of any potentially identifiable images or data included in this article.

## Author Contributions

All authors listed have made a substantial, direct and intellectual contribution to the work, and approved it for publication.

## Conflict of Interest

The authors declare that the research was conducted in the absence of any commercial or financial relationships that could be construed as a potential conflict of interest.
